# Effect of infant feeding practices on iron status in a cohort study of Bolivian infants

**DOI:** 10.1186/s12887-018-1066-2

**Published:** 2018-03-12

**Authors:** Rachel M. Burke, Paulina A. Rebolledo, Anna M. Aceituno, Rita Revollo, Volga Iñiguez, Mitchel Klein, Carolyn Drews-Botsch, Juan S. Leon, Parminder S. Suchdev

**Affiliations:** 1 Department of Epidemiology, Rollins School of Public Health, Emory University, Claudia Nance Rollins Building, 1518 Clifton Rd. NE, Atlanta, GA 30322 USA; 2 Hubert Department of Global Health, Rollins School of Public Health, Emory University, Atlanta, GA USA; 3 Emory School of Medicine, Atlanta, GA USA; 4 Servicio Departamental de Salud, La Paz, Bolivia; 5 Instituto de Biotecnología y Microbiología, Universidad Mayor de San Andrés, La Paz, Bolivia; 6 Nutrition Branch, Centers for Disease Control & Prevention, Atlanta, GA USA

**Keywords:** Micronutrients, Iron deficiency, Global nutrition, Infant nutrition, Global health, Breastfeeding

## Abstract

**Background:**

Iron deficiency (ID) is the most common micronutrient deficiency worldwide, with potentially severe consequences on child neurodevelopment. Though exclusive breastfeeding (EBF) is recommended for 6 months, breast milk has low iron content. This study aimed to estimate the effect of the length of EBF on iron status at 6 – 8 months of age among a cohort of Bolivian infants.

**Methods:**

Mother-infant pairs were recruited from 2 hospitals in El Alto, Bolivia, and followed from one through 6 – 8 months of age. Singleton infants > 34 weeks gestational age, iron-sufficient at baseline, and completing blood draws at 2 and 6 – 8 months of age were eligible for inclusion (*N* = 270). Ferritin was corrected for the effect of inflammation. ID was defined as inflammation-corrected ferritin < 12 μg/L, and anemia was defined as altitude-corrected hemoglobin < 11 g/dL; IDA was defined as ID plus anemia. The effect of length of EBF (infant received only breast milk with no other liquids or solids, categorized as < 4, 4 – 6, and > 6 months) was assessed for ID, IDA, and anemia (logistic regression) and ferritin (Fer) and hemoglobin (Hb, linear regression).

**Results:**

Low iron status was common among infants at 6 – 8 months: 56% of infants were ID, 76% were anemic, and 46% had IDA. EBF of 4 months and above was significantly associated with ID as compared with EBF <  4 months (4 – 6 months: OR 2.0 [1.1 – 3.4]; > 6 months: 3.3 [1.0 – 12.3]), but not with IDA (4 – 6 months: OR 1.4 [0.8 – 2.4]; > 6 months: 2.2 [0.7 – 7.4]), or anemia (4 – 6 months: OR 1.4 [0.7 – 2.5]; > 6 months: 1.5 [0.7 – 7.2]). Fer and Hb concentrations were significantly lower with increasing months of EBF.

**Conclusions:**

Results suggest a relationship between prolonged EBF and ID, but are not sufficient to support changes to current breastfeeding recommendations. More research is needed in diverse populations, including exploration of early interventions to address infant IDA.

**Electronic supplementary material:**

The online version of this article (10.1186/s12887-018-1066-2) contains supplementary material, which is available to authorized users.

## Background

Iron deficiency (ID) is the most common micronutrient deficiency, affecting an estimated 40% of children under 5 and 38% of pregnant women globally [1]. If uncorrected, ID can progress into iron deficiency anemia (IDA); ID and IDA have been associated with potentially irreversible deficits in cognitive development in infants and children [2, 3].

Infants have high iron needs due to their rapid growth [4, 5]. Young infants are thought to be protected from ID via their birth iron stores, which are largely accumulated during the last trimester of gestation and depleted through the first 4 – 6 months of life [6, 7]. Yet ID has been identified even in very young populations of healthy infants [8–12], raising questions about optimal infant and young child feeding and supplementation practices [13]. The World Health Organization (WHO) recommends exclusive breastfeeding (EBF; defined as no foods or liquids other than breast milk and supplements or medications) for infants up to 6 months of age, due to the excellent nutritional content and demonstrated immunological benefits of breast milk [14, 15]. However, although the iron in breast milk is highly bioavailable, it is present in only small amounts [8, 13, 16], prompting discussion as to whether EBF should be recommended only to 4 months of age (as in earlier recommendations) as opposed to 6 months of age [15, 17]. In multiple studies, the length of EBF or predominant breastfeeding (PRBF) has been associated with poorer iron status [18–23], while in other studies, EBF has not been associated with iron status [24, 25], or has been associated with some markers of iron status, but not others [26, 27].

Much of the existing literature on this topic employs cross-sectional designs, limiting the ability to understand longitudinal patterns. Further, very few studies account for the effect of inflammation, which can transiently increase ferritin, the most sensitive marker of iron status (and the marker recommended by WHO and the Centers for Disease Control and Prevention [CDC]) [28, 29]. Although studies exist in developing countries, few were conducted in high-altitude settings, [23] where iron needs may be higher [30], or in settings with high coverage of infant iron supplementation.

In the present study, we aim to estimate the effects of length of EBF on iron deficiency (ID), anemia, and iron deficiency anemia (IDA) in a cohort of healthy infants followed from birth through 6 – 8 months of age, while using a previously described and employed method to adjust for the effect of inflammation on iron biomarkers [31]. Previous work in this population identified a high prevalence of ID, anemia, and IDA in infants and young toddlers, despite supplementation programs targeting mothers and children [31]. The present study will provide information on the impact of the length of EBF on iron status, in a developing country with a high burden of malnutrition [32] and a national micronutrient supplementation program [33].

## Methods

### Study population and design

Data for the present study were drawn from the *Nutrición, Inmunología, y Diarrea Infantil* (NIDI) study, the primary aim of which was to assess differences in infant immune response to the rotavirus vaccine (Rotarix®), by nutritional status. In brief, 461 healthy infants (2 – 4 weeks of age) and their mothers were recruited from 2 hospitals in El Alto, Bolivia (altitude 4000 m), during well-child or vaccination visits. Bolivia has a national supplementation program providing families with 60 sachets of Chispitas (multiple micronutrient powder [MNP] containing 12.5 mg of iron as ferrous fumarate, 5 mg zinc, 300 μg vitamin A, 30 mg vitamin C and 180 μg folic acid per sachet) every 6 months for all infants 6 to 59 months of age; preterm infants are also recommended to receive iron drops during the first 2 – 6 months of life [33]. The population of El Alto is primarily urban and largely indigenous; socioeconomic resources are typically low [31]. Exclusion criteria included infant illness at recruitment, suspicion of immunodeficiency (e.g., HIV), congenital malformations, and maternal inability to speak and understand Spanish or Aymara. Recruitment took place May 2013 – March 2014, and infant-mother dyads were followed through 6 – 10 months of age, with final data collected in March 2015. Hospital visits occurred at target dates of 1, 2, 3, 4, and 6 – 8 months of age, with blood drawn at the 2nd and 5th visits (at approximately 2 and 6 – 8 months, respectively). For the present study, iron status was assessed using the second blood draw, corresponding to the age at which iron stores begin to show depletion. Singleton infants > 34 weeks gestational age, with non-missing data on outcomes and covariates, and who were not ID at 2 months (*N* = 270) were eligible for analyses, since multiples and early preterm infants may have different feeding practices [34] and may be more vulnerable to ID compared to term, singleton infants [5].

### Ethical approval

The protocol and instruments for this study were approved by the Emory University IRB (IRB00056127) and the Bolivian “Comité de Etica de la Investigación” (Research Ethics Committee). Mothers provided written informed consent in Spanish or Aymara.

### Laboratory analysis and definitions of iron status

Venous blood was collected (1 mL) from mothers and infants using zinc-free equipment. Hemoglobin (Hb) was measured at point-of-care using a HemoCue® photometer. Plasma was analyzed by sandwich ELISA for ferritin and two markers of inflammation: C-Reactive Protein (CRP; limit of detection [LOD]: 0.5 mg/L) and alpha(1)-acid-glycoprotein (AGP; LOD: 0.1 g/L). [35] Hb was adjusted for the high altitude (3500 – 4000 m) of El Alto and surroundings [36]. Anemia was defined as adjusted Hb < 11 g/dL, based on WHO guidelines [37]. ID was defined as ferritin < 12 μg/L [29]. IDA was defined as ID plus anemia. In all cases, ferritin was adjusted for the effect of inflammation (CRP and AGP) using a linear regression method described in detail elsewhere [31, 38]. Briefly, ferritin (log-transformed to meet normality assumptions) was modeled as a function of continuous CRP and AGP (also log-transformed to improve model fit), and estimated coefficients of AGP and CRP were then used to adjust ferritin back to a counterfactual value under non-inflammation conditions. The relationship of inflammation to iron biomarkers and the impact of adjustment on ID prevalence in this study population are presented elsewhere and therefore will not be elaborated here [31].

Mothers and infants were referred for anemia according to Bolivian guidelines (mothers < 13.7 g/dL; infants < 10.9 g/dL), and infants were referred for stunting (length-for-age Z score < − 2) or wasting (weight-for-length Z score < − 2) at any visit.

### Data collection

Sociodemographic data was collected by trained Bolivian interviewers at the first study visit via questionnaire. Birth weight was corroborated by health card in 60% of cases, but was not significantly different by maternal report. At each visit, interviewers collected data on recent infant morbidities and feeding practices, including whether the infant was breastfed within the last 24 h, and whether the infant had ever received any non-breast milk liquids (e.g., formula, cow’s milk, water) or CF. The predominantly used formula brand was iron fortified, but the predominantly reported CF were not iron fortified.

### Variable definitions and statistical analysis

EBF was based on maternal recall of feeding practices through each visit and calculated from the infant age at the last visit where the infant was reported to have been fed only breast milk, without ever having received any non-breast milk liquids or CF. CF was defined as any semi-solid or solid food (e.g., yogurt, mashed vegetables). Given past and present WHO recommendations on feeding practices [14, 15], EBF was categorized as follows: < 4 months, 4 – < 6 months, and ≥6 months. Other variables relating to feeding practices were considered to be potential intermediates and therefore not included.

Potential covariates were informed based on a conceptual diagram (Additional file 1: Figure S1) and selected based on bivariate associations with the outcome and the exposure. Initial models included infant age at blood draw (dichotomized as ≥7 months vs. 6 months), birth weight, sex, maternal age (dichotomized as < 20 years vs. ≥ 20 years), maternal education (university education, secondary education, primary education, or less than primary, later dichotomized to university education vs. less than university education), maternal relationship status (single vs. married or cohabiting), maternal employment, cell phone ownership, and roof construction materials. Iron supplementation was not included in models, as it was not significantly related to outcome or exposure in bivariate analysis (perhaps due to the short time period between receipt and assessment). Chispitas were not included in final models because their receipt implies CF; further, their use was not significantly associated with outcomes in bivariate analysis. Final models were reduced to prioritize parsimony and consistency of covariates across outcomes and exposures, while controlling for confounding (maintaining exposure effect estimates within 10% of the initial fully adjusted models) [39].

Linear regression was used to assess relationships of exposures to continuous ferritin and Hb (log-transformed to meet normality assumptions and corrected for inflammation [ferritin] or altitude [Hb]). Binary logistic regression was used to assess relationships between the exposures and the categorized outcomes. All models were tested for collinearity using Variance Decomposition Proportions (VDPs) and Condition Indices (CIs); no problems were identified. Wald chi-square tests were used to assess significance except for EBF categories, where Likelihood Ratio tests were used; *P* <  0.05 was considered statistically significant. Effect modification was not assessed. Data were cleaned and analyzed using SAS v9.4 (Cary, NC) and the R Environment for Statistical Computing [40].

## Results

### Characteristics of the study sample

**Fig. 1:**
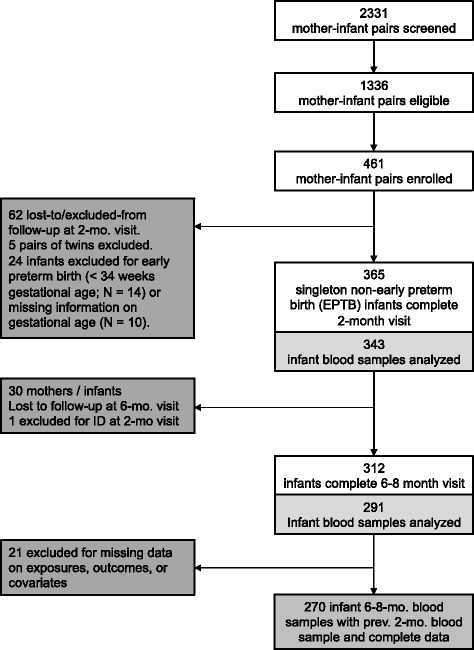
Participant Flow. Of 2331 screened mother-infant pairs, 1336 were eligible for the parent study and 461 enrolled. A total of 343 singleton, non-early preterm infants provided samples at 2 months, with 291 infants iron-sufficient at baseline giving samples at 6 – 8 months, and 270 having complete data

Out of 451 singletons enrolled in the parent study, 365 completed initial study requirements (first dose of Rotarix® vaccine and blood draw at 2 months of age) and were of eligible gestational age (> 34 weeks). Of these, 30 were lost to follow-up before the second blood draw, and one was ID at the initial blood draw. Of the remaining 312 infants, 291 had data for both the first and second blood draws; however, 21 were missing data on exposures or covariates. The study population for the present analysis thus included 270 singleton infants (Fig. 1).

The median age of infants at the time of assessment was nearly 7 months (SD 1 month). Infants were fairly evenly distributed in terms of gender, nearly one third were born via caesarean section, and one twentieth were low birth weight (Table 1). Mothers had a mean age of 26 years, one-half were first-time mothers, one-quarter were employed, and most had at least a secondary education.

**Table 1:** Characteristics of the Study Sample, El Alto, Bolivia (*N* = 270)

	Frequency or Mean (±SD)	Percent
Infant Characteristics		
Age (months) at blood draw^a^	6.7 ± 0.9	–
Male	142	52.6
Caesarean section	81	30.0
Late preterm (34 - 37 weeks gestational age)	35	13.0
Low birth weight (< 2500 g)	15	5.6
Inflammation (elevated CRP or AGP)^b^	55	20.4
Maternal Characteristics		
Maternal age (years)	26.0 ± 6.5	–
Primipara	127	47.0
Took > 1 month of prenatal iron supplementation^**c**^	138	58.0
Maternal employment	73	27.0
* Maternal Education*		
Completed university	31	11.5
Completed secondary only	142	52.6
Completed primary only	70	25.9
Did not complete primary	27	10.0
Infant Feeding and Supplementation		
Ever breastfed	269	99.6
Number of months of exclusive breastfeeding^d^	3.1 ± 2.3	–
Exclusively breastfed^d^ until 6 months	78	28.9
Exclusively breastfed^d^ until 4 months	144	53.3
Ever received formula	138	53.9
Received semi-solid foods in the previous 24 h	223	82.6
Has taken Chispitas^e^	50	18.6
Has taken iron drops^f^	21	7.8
Iron Status Indicators		
Iron deficiency (ID) ^*g*^	151	55.9
Iron deficiency anemia (IDA)^h^	125	46.3
Anemia^i^	204	75.6
Hb (g/dL)	12.7 ± 1.3	

^a^152 infants (56%) were ≥7 months at blood draw. ^b^Defined as AGP > 1 g/L or CRP > 5 mg/L. ^**c**^*N* = 231 due to missing data. ^d^Defined as infant received no semi-solid foods or non-breast milk liquids until reaching 6 months of age. Based on maternal recall. 21 infants were exclusively breastfed beyond 6 months of age. ^e^Multiple micronutrient powder (MNP) sachets containing 12.5 mg of iron as ferrous fumarate. ^f^Recommended for preterm infants 2 – 6 months of age; of late preterm infants, 8 (22.9%) reported having taken iron drops. ^*g*^Defined as inflammation-corrected ferritin < 12 μg/L, see Methods. ^h^Defined as iron deficiency plus anemia. ^i^Defined as altitude-corrected hemoglobin < 11 g/dL, see Methods

### EBF, complementary feeding, and iron status

Although nearly all infants had been breastfed at some point in their lives, only 53% were EBF until at least 4 months, and 29% EBF until 6 months of age; the mean length of EBF was 3 months (Table 1). At the time of the blood draw, 83% of infants had received some semi-solid food in the previous day. Nearly 20% of infants had taken Chispitas (multiple micronutrient powder [MNP] supplements containing 12.5 g iron as ferrous fumarate per daily sachet, Table 1). Low iron status was common: 56% of infants were ID, 76% were anemic, and 46% had IDA (Table 1); 61% of anemic infants were also ID (data not shown).

### Associations of feeding practices with iron status indicators

#### Effect of length of EBF on continuous outcomes

Given WHO recommended practices [14], we assessed the impact of EBF categorized as < 4 months, 4 – < 6 months, and ≥6 months on ferritin and Hb. Adjusted linear regression models demonstrated significant relationships: both ferritin and Hb decreased as the number of months of EBF increased, though the effect on ferritin was much larger (ferritin decreased by 16% for infants EBF 4 – 6 as compared to < 4 months, while Hb decreased only by 3% for the same comparison [Table 2]).

**Table 2:** Association of Length of Exclusive Breastfeeding with Ferritin and Hemoglobin^a^ (*N* = 270)

	Ferritin			Hb		
	Percent Difference from Referent	CI	*P* value**	Percent Difference from Referent	CI	*P* value**
*Length of exclusive breastfeeding* ^*b*^						
< 4 months (ref.; *N* = 93)	0.0	–	0.043	0.0	–	0.039
4 - < 6 months (*N* = 156)	−16.3	(−31.9, 2.9)	–	−2.7	(−5.1, −0.3)	–
≥ 6 months (*N* = 21)	−38.2	(−62.5, −4.9)	–	−4.4	(−9.1, 0.7)	–
*Covariates*						
Infant ≥7 months old at blood draw (vs. 6 mo.)	−22.2	(−30.4, − 13.0)	< 0.0001	−1.6	(− 2.9, − 0.3)	0.017
Male sex (vs. female)	48.9	(21.3, 82.7)	0.0002	4.1	(1.6, 6.6)	0.001
Birth weight^c^	25.9	(13.9, 39.1)	< 0.0001	2.9	(1.7, 4.1)	< 0.0001
Maternal employment (vs. none)	34.1	(6.9, 68.3)	0.012	1.7	(−1.0, 4.4)	0.23
Mother has completed university education (vs. lower levels of education or no education)	51.0	(9.6, 108.1)	0.012	1.1	(−2.7, 5.0)	0.58

^a^Ferritin and Hb log-transformed to meet normality assumptions. Percent change calculated based on back-transformed values. **Wald Chi-Square tests. ^b^Defined as infant received no semi-solid or solid foods or non-breastmilk liquids until reaching 4 months of age. Based on maternal recall. ^c^500g increase

#### Effect of length of EBF on dichotomized outcomes

**Fig. 2:**
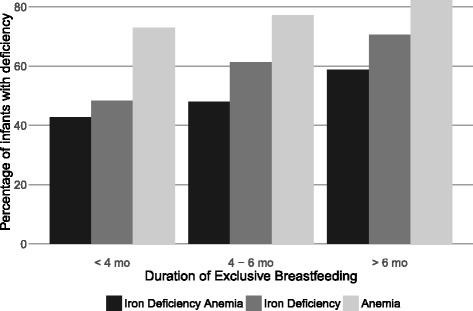
Prevalence of Iron Deficiency and Iron Deficiency Anemia by Duration of Exclusive Breastfeeding (*n* = 270). The prevalence of iron deficiency was increased among infants who had longer durations of exclusive breastfeeding. A similar but less pronounced trend was noted for iron deficiency anemia

We also assessed the impact of EBF (categorized as above) on ID, anemia, and IDA. Deficiencies tended to be higher with increased length of EBF (Fig. 2). In multivariable models, longer EBF was significantly associated with ID, but not with IDA or anemia, although IDA patterns were similar to ID. Odds of all outcomes were also significantly increased among lower-birth-weight infants and males (vs. females). Odds of ID and IDA were significantly lower among infants whose mothers were employed as well as among infants whose mothers had completed a university education. Older infants had significantly higher odds of IDA and anemia. (Table 3.)

**Table 3:** Association of Exclusive Breastfeeding to 6 months with Iron Deficiency (ID), Iron Deficiency Anemia (IDA), and Anemia^a^ (N = 270)

	ID			IDA			Anemia		
	OR	95% CI	P value**	OR	95% CI	*P* value**	OR	95% CI	*P* value**
*Length of exclusive breastfeeding* ^*b*^									
< 4 months (ref.; *N* = 93)	1.00	–	0.015	1.00	–	0.25	1.00	–	0.57
4 - < 6 months (*N* = 156)	1.99	(1.17, 3.43)	–	1.39	(0.82, 2.40)	–	1.36	(0.74, 2.52)	–
≥ 6 months (*N* = 21)	3.25	(1.01, 12.27)	–	2.23	(0.72, 7.35)	–	1.49	(0.41, 7.19)	–
*Covariates*									
Infant ≥7 months old at blood draw (vs. 6 mo.)	1.58	(0.94, 2.70)	0.088	1.89	(1.12, 3.22)	0.017	2.45	(1.32, 4.70)	0.005
Male sex (vs. female)	1.81	(1.07, 3.11)	0.029	2.48	(1.46, 4.29)	0.0009	2.73	(1.49, 5.11)	0.001
Birth weight^c^	0.59	(0.45, 0.77)	0.0001	0.57	(0.43, 0.75)	0.0001	0.61	(0.46, 0.83)	0.0001
Maternal employment (vs. none)	0.51	(0.28, 0.91)	0.023	0.52	(0.28, 0.94)	0.033	0.67	(0.35, 1.31)	0.23
Mother has completed university education (vs. lower levels of education or no education)	0.32	(0.13, 0.75)	0.009	0.36	(0.15, 0.85)	0.023	1.57	(0.57, 5.14)	0.41

^a^Iron Deficiency defined as inflammation-corrected ferritin < 12 μg/L, see Methods. Anemia defined as altitude-corrected hemoglobin < 11 g/dL, see Methods. Iron Deficiency Anemia defined as Iron Deficiency plus Anemia. **Wald Chi-Square tests. ^b^Defined as infant received no semi-solid or solid foods or non-breastmilk liquids until reaching 6 months of age. Based on maternal recall. ^c^500g increase

## Discussion

ID, anemia and IDA were common among this cohort of primarily breastfed, healthy Bolivian infants. Analyses demonstrated a significant inverse association between continuous ferritin and months of EBF, as well as a significant positive association between ID and months of EBF. Results for IDA demonstrated similar patterns to ID analyses, but were attenuated and non-significant. Although there was a small significant association between length of EBF and Hb, there was not a significant association between length of EBF and anemia. The effect of inflammation was accounted for in all models of ID and IDA, but the ferritin-inflammation relationships have already been elaborated in a previous publication and therefore are not discussed here [31].

While our findings suggest a potential relationship between feeding practices—particularly the duration of EBF—and iron status, they do not clearly support any change in current recommendations of 6 months of EBF. The association of continuous ferritin with months of EBF is consistent with several other studies in diverse settings (two RCTs—in Honduras and Iceland—as well as a cohort study in Mexico) [18, 19, 24, 41] in addition to biological understanding that breast milk is comparatively low in iron versus formula or complementary foods [8, 13, 16]. The lack of significant associations between length of EBF and IDA in our study may reflect a lack of power (a post-hoc power calculation for the effect of EBF to ≥4 months on IDA showed < 40% power to detect an OR of 1.5), or it may reflect the influence of anemia (less associated with EBF) on the development of IDA. Given that not all studies may be able to collect information on inflammatory biomarkers, we conducted an additional sensitivity analysis testing the effect of feeding practices on uncorrected ferritin; this showed very similar results.

The results of our study do not support any change in recommended feeding practices for the prevention of anemia. While continuous Hb was significantly inversely associated with the length of EBF, there was no significant relationship of feeding practices to anemia. The finding of a significant relationship between feeding practices and Hb is similar to findings in an RCT of Honduran infants [41] as well as cohort studies of Mexican [19] and Nepali infants [23]; these populations had a high prevalence of breastfeeding (but lower EBF), similar to our population, although the prevalence of anemia was much lower in the Mexican infants [19] as compared to the Honduran infants [41], Nepali infants [23] or to our own population. Two cohort studies—one in Bangladesh [24] and one in Iceland [18]—found no significant associations of Hb with feeding practices; however, it is worth noting that the prevalence of LBW was extremely high in the Bangladeshi infants (30%) [41], while the Icelandic infants had much higher birth weight as well as Hb levels [18], potentially limiting our ability to compare results to these studies. The fact that a large proportion of the anemic infants were not ID may suggest that more important causes of anemia exist in this population (potentially including the altitude or lack of folate or Vitamin B-12 in the infant diets); however, this issue may also reflect a need for a more valid Hb cut-off. Unfortunately, this study was not designed to identify non-iron-related causes of anemia.

The present study has several strengths. A primary strength is the adjustment for the effect of inflammation on iron biomarkers, using two markers of inflammation to capture varying stages of the acute phase response [31]. The vast majority of previous studies, if they accounted for inflammation at all, have only done so by excluding infants with high CRP [18, 24, 41]. Another strength is the longitudinal design, enabling us to follow infants almost from birth while frequently collecting data on feeding practices. Further, our population of healthy, primarily breastfed infants in a developing country allows us to assess the effect of recommended feeding practices on iron status in a low-resource population. This is also one of few studies to simultaneously assess ID, anemia, IDA, ferritin, and Hb. However, the study also has some limitations. Maternal recall of feeding practices may be imperfect. Although data on feeding practices was collected at each visit, there were at least 2 months between the last and the penultimate visit, introducing the possibility of misclassification. However, it is reassuring that these visits corresponded to roughly 4 – 5 and 6 – 8 months of age, meaning that the vast majority of infants would already have completed the ages corresponding to our EBF cut-offs. Further, the length of EBF was not related to the time between these two visits, and all models controlled for age at blood draw (related to time between visits), again mitigating the possibility of differential misclassification. Further, no infant changed EBF categories if feeding data from a subsequent visit was used. Although there was a low participation rate (mainly due to lack of interest or refusal of blood draw), characteristics of enrolled mothers and infants in the present study were very similar to those in a pilot study by our same group in the same hospitals but not requiring blood draws (data not shown). Although these results may be generalizable to other developing country and high-altitude Andean populations, they may not generalize to settings with a high prevalence of other causes of anemia (such as malaria or HIV). Anemia results may not be generalizable to lower-altitude settings.

## Conclusions

This study suggested a relationship between duration of EBF and iron status, with higher odds of ID among infants EBF for 4 months and longer as opposed to less than 4 months. However, the results are insufficient to support any changes to current recommendations of 6 months of EBF. More research in diverse populations, while controlling for the effect of inflammation, would help to contextualize these results. Nonetheless, the high prevalence of ID, IDA, and anemia, as well as the relationship of iron status to birth weight and feeding practices, suggest a need for additional research to assess the role of early iron supplementation (to be implemented prior to the initiation of CF) and other preventive interventions in lower birth-weight and other vulnerable populations.

## Additional file

Additional file 1: Conceptual diagram of the relationship between the length of exclusive breastfeeding and infant iron status at 6 - 8 months of age. (PDF 312 kb)

## Abbreviations

AGP: alpha(1)-acid glycoprotein
APR: Acute Phase Response
BI: body iron
CRP: C-reactive protein
Fer: Ferritin
Hb: hemoglobin
ID: iron deficiency
IDA: iron deficiency anemia
MNP: multiple micronutrient powder
sTFR: soluble transferrin receptor
