# Supplementary figures and images for: Effect of infant feeding practices on iron status in a cohort study of Bolivian infants
Source: BMC Pediatr. 2018 Mar 12;18:107. doi: 10.1186/s12887-018-1066-2 (PMC5848561; doi:10.1186/s12887-018-1066-2)

## Legend

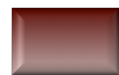

Outcome

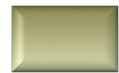

Exposure

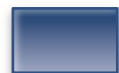

Measured covariate

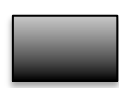

Unmeasured  
covariate

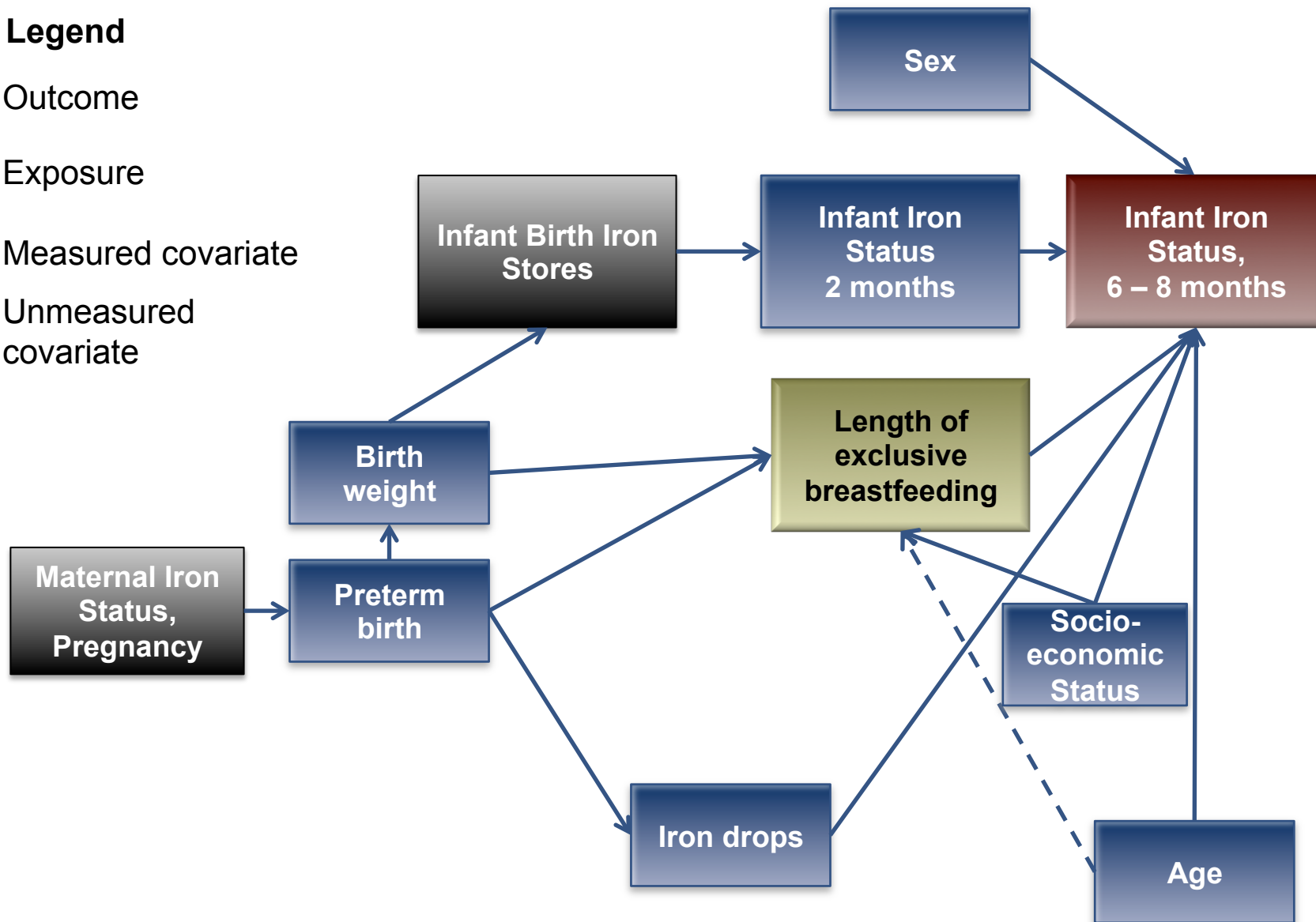

Supplement: Supplementary file 1 — Conceptual diagram of the relationship between the length of exclusive breastfeeding and infant iron status at 6 - 8 months of age. (PDF 312 kb) [file 12887_2018_1066_MOESM1_ESM.pdf]
